# Hierarchical Organization of Corticothalamic Projections to the Pulvinar

**DOI:** 10.1093/texcom/tgaa030

**Published:** 2020-07-07

**Authors:** Reza Abbas Farishta, Denis Boire, Christian Casanova

**Affiliations:** 1 École d’optométrie, Université de Montréal, Québec, Canada; 2 Département d’anatomie, Université du Québec à Trois-Rivières, Québec, Canada

**Keywords:** area 21a, corticothalamic, pulvinar, transthalamic pathways, visual hierarchy

## Abstract

Signals from lower cortical visual areas travel to higher-order areas for further processing through cortico-cortical projections, organized in a hierarchical manner. These signals can also be transferred between cortical areas via alternative cortical transthalamic routes involving higher-order thalamic nuclei like the pulvinar. It is unknown whether the organization of transthalamic pathways may reflect the cortical hierarchy. Two axon terminal types have been identified in corticothalamic (CT) pathways: the types I (modulators) and II (drivers) characterized by thin axons with small terminals and by thick axons and large terminals, respectively. In cats, projections from V1 to the pulvinar complex comprise mainly type II terminals, whereas those from extrastriate areas include a combination of both terminals suggesting that the nature of CT terminals varies with the hierarchical order of visual areas. To test this hypothesis, distribution of CT terminals from area 21a was charted and compared with 3 other visual areas located at different hierarchical levels. Results demonstrate that the proportion of modulatory CT inputs increases along the hierarchical level of cortical areas. This organization of transthalamic pathways reflecting cortical hierarchy provides new and fundamental insights for the establishment of more accurate models of cortical signal processing along transthalamic cortical pathways.

## Introduction

The receptive field properties of neurons in sensory cortices undergo major transformation as they progress along ascending pathways (Stone et al. 1979). This growing complexity is generally considered to originate from processing between anatomically and functionally organized cortical areas classified in a hierarchical manner according to the laminar pattern of their originating and terminating projections (Felleman and Van Essen 1991; Markov et al. 2014).

While models using this classification of cortical processing have greatly advanced our understanding of the visual system (Scannell et al. 1995), they remain incomplete as they restrict complex visual processing to direct cortico-cortical pathways only while it is known that all visual cortical areas are also reciprocally connected to the main extra-geniculate thalamic nucleus, the pulvinar, providing them monosynaptic transthalamic pathways between areas of the neocortex (Shipp 2003; Theyel et al. 2010; Sherman and Guillery 2011; Sherman 2016).

The role of the associated trans-thalamic pathways remains unclear, and it is still unknown how their functions differ from that of the corresponding direct cortico-cortical pathways (Casanova et al. 2001; Sherman 2017). Studies suggest that these pathways can be used to facilitate the cortical flow of information (Theyel et al. 2010), modulate contrast sensitivity (Cortes and van Vreeswijk 2012; de Souza et al. 2019), and subtend attentional processes (Saalmann et al. 2012; Ni et al. 2016; Roth et al. 2016; Zhou et al. 2016).

One way to increase our understanding of these pathways is to determine whether they parallel the hierarchical organization of the direct cortico-cortical connections. In this context, 2 types of corticothalamic (CT) axons connecting cortical areas to the pulvinar have been identified based on their morphology. Type I axons are thin, possess long thin branches with occasional *en passant* swellings, and show highly divergent and quasi-linear segments, while type II axons possess thicker axons, larger terminals ranging between lone singletons and more complex flowery forms of rosettes and terminate in one or several small compact arbors showing complex arborization (Ojima et al. 1996; Vidnyanszky et al. 1996; Feig and Harting 1998; Guillery et al. 2001; Huppe-Gourgues et al. 2006; Huppe-Gourgues et al. 2019). According to the “driver/modulator” theory of glutamatergic pathways involving the thalamus and the cortex, type II terminals (driver) are believed to be the main carrier of sensory information, while type I (modulator) are assumed to fine-tune ongoing activity (Sherman and Guillery 1998).

Studies have revealed that the proportion of type I and type II cortico-pulvinar terminals varies between cortical areas (Huppe-Gourgues et al. 2006; Huppe-Gourgues et al. 2019). For instance, in the cat pulvinar complex, the vast majority of axons coming from V1 has type II terminals, while those from the posteromedial lateral suprasylvian (PMLS) cortex and the anterior ectosylvian sulcus (AES) mainly exhibit type I terminals (Huppe-Gourgues et al. 2019). These data suggest that the nature of cortical projections to extra-geniculate thalamic nuclei varies with the hierarchical level of visual areas.

To test the hypothesis that, like their cortico-cortical counterpart, transthalamic pathways may also reflect the organization of the hierarchy of visual areas, we charted the distribution of projections from area 21a, located along the ventral visual stream and mainly involved in pattern and form recognition and visual cognitive processes (Dreher et al. 1993; Dreher et al. 1996; Villeneuve et al. 2009). These data were compared with those acquired for 3 other visual areas of different hierarchical order from our previous study (Huppe-Gourgues et al. 2019): area 17 (V1), the PMLS (located at a lower rank than area 21a), and the AES, (located at a higher rank than area 21a) (Scannell et al. 1999). The objectives of this study were 3-fold: 1) to determine the morphology of CT projections from area 21a, 2) to compare these projections with the known type II projections from area 17 (Guillery et al. 2001; Huppe-Gourgues et al. 2006) and those from cortical areas of the dorsal stream (PMLS and AES from Huppe-Gourgues et al. 2019), and 3) to test the hypothesis that the organization of CT projections reflects the cortical hierarchy of visual areas.

## Materials and Methods

### Animal Preparation and Injections

Animals were treated in accordance to the regulations of the Canadian Council for the Protection of Animals, and the experimental protocols were approved by the “Comité de déontologie de l”expérimentation sur les animaux” of the Université de Montréal. Five normally pigmented adult cats were used in this study. Four were used to study the morphology of area 21a CT projections (*n* = 4 injections in area 21a). For comparison purposes, one cat (*n* = 1) received an injection in area 17 (*n* = 1) and used as a control to replicate the very well-documented type II striate projections to the LP-pulvinar (Guillery et al. 2001; Huppe-Gourgues et al. 2006). Preoperative anti-inflammatory agents (metacam s.c. 0.1 mg/kg) and antibiotics (tribrissen 24% s.c. 0.13 mL/kg) were administered 24 h before surgical procedures. Twenty minutes before surgery, atropine (0.1 mg/kg s.c.) and Atravet (0.05 mg/kg s.c.) were given to the animal. Anesthesia was induced with a mixture of 5% isoflurane in 60% N_2_O and 25% O_2_ and maintained with 2% Isoflurane added to the same gaseous mixture. Animals were positioned in a stereotaxic apparatus. A local anesthetic (lidocaine hydrochloride 2%) was injected subcutaneously, and a craniotomy was performed over the injection site. During all surgical procedures, animals were maintained at 38 °C, and heart rate, end-tidal CO_2_, blood O_2_ saturation, and blood pressure were closely monitored.

Area 21a craniotomies were performed according to Horsley–Clarke (H-C) coordinates 6–12 mm lateral to the midline and 0–8 mm posterior to the interaural plane. For area 17, the craniotomy was performed according to Horsley–Clarke (H-C) coordinates 0–4 mm lateral to the midline and 0–9 mm posterior to the interaural plane. Borosilicate pipettes (1.5 mm external diameter) were pulled to obtain a tip ranging between 20 and 40 μm. Biotinylated dextran amines (BDA 10 kDa) were injected (Molecular Probes, Thermo Fisher Scientific, MA, USA) in area 21a by iontophoresis using a positive DC current (7 s on/off cycle 7 μA) for 25 min at depths of 1.2 and 0.8 mm in order to target all cortical layers. In all cases, the injection sites never encroached the underlying white matter. Craniotomies were sealed with acrylic bone cement, and the wounds were sutured in anatomical layers. Anti-inflammatory and antibiotic treatments were administered pre- and postoperatively, and analgesic was applied for 72 h following surgery (Metacam/Boehringer-Ingelheim.ca; 0.01 mg/kg bid).

### Tissue Processing

Ten to fourteen days after the cortical injections, animals received an overdose of sodium pentobarbital (80 mg/kg; IP) and were perfused with phosphate-buffered 0.9% saline (PBS: 0.1 M, pH 7.4) followed by phosphate buffered 4% paraformaldehyde. Brains were blocked stereotaxically, removed from the cranium, postfixed overnight in the same fixation solution at 4 °C, cryoprotected in 30% sucrose in 0.1 M phosphate buffer (pH 7.4), and frozen until processed. The fixed brains were cut into 40 μm-thick coronal sections and collected in PBS. After preincubation in 2.5% bovine serum albumin and normal goat serum 2% in PBS (0.01 M PB with 0.9% NaCl, pH 7.4) for 30 min, BDA was visualized with avidin-biotin-peroxidase complex (ABC; Vectastain ABC Elite kit; Vector, Burlingame, CA). Following buffer washes, sections were reacted with nickel-intensified diaminobenzidine (0.5% nickel, 0.035% diaminobenzidine and 0.002% H_2_O_2_) for 10 min (Hsu and Soban 1982). After PBS washes, sections were mounted on slides, dehydrated, mounted with Depex, and cover-slipped. Adjacent sections were processed for acetylcholinesterase (AChE) histochemistry for the identification of cytoarchitectonic boundaries between the lateral (LPl) and medial (LPm) subdivisions of the lateral posterior thalamus of the LP-pulvinar complex (Graybiel and Berson 1980). Sections were incubated for 6 h in an aqueous solution with 50 mM sodium acetate, 4 mM copper sulfate, 16 mM glycine, 4 mM *S*-acetylthiocholine, and 86 μM ethopropazine adjusted to pH 5. Sections were rinsed in water and reacted for 10 min in a 1% aqueous solution of sodium sulfite and subsequently fixed in 4% paraformaldehyde for 2 h. The location of area 21a was confirmed by staining slides adjacent to the injection site with the monoclonal antibody SMI 32 known to reveal the parcellation of visual cortical areas (van der Gucht et al. 2001). Briefly, free-floating tissue sections were preincubated for 1 h in Tris-buffered saline containing 0.1% Triton X-100 and 5% normal goat serum. Sections were then incubated overnight in Tris-buffered saline containing 0.1% Triton X-100, 5% normal goat serum, and primary monoclonal antibody (mouse monoclonal anti-SMI32 specific for nonphosphorylated Neurofilament H, (Catalog number 801701, 1:2000, Biolegend, CA). On the following day, immunoreactivity was revealed using a Vectastain ABC kit (Vector Laboratories, Burlingame, CA) and chromogen 3,3′-diaminobensidine with peroxide.

### Analysis

For stereological analysis in the pulvinar, evenly spaced sections (1 of every 5 sections) were selected and projection fields were outlined under a 10× objective using a microscope (DMR, Leica) equipped with a three-axis computer-controlled stepping motor system coupled to a computer and to a color Optronix CCD camera and driven by the Neurolucida software (MBF Biosciences, Williston, VT, USA). Contours of each thalamic area presenting anterogradely labeled axons and terminals were charted. These contours were superimposed on the images of adjacent AChE-reacted sections and resized for shrinkage differences between the AChE and BDA sections. This allowed CT projections to be assigned to either the LPl or LPm.

In order to provide an unbiased mapping of the distribution of axon terminals from areas 21a and 17 to the pulvinar and their size frequency, a systematic stereological random sampling of these projection fields was performed using the Stereo Investigator software (MBF Bioscience). Projection fields in which anterograde labeling was observed were sampled under a 100× oil immersion lens (HC PL APO 100×/1.25 PH3, Leica, Germany) using the optical fractionator workflow (in Stereo Investigator) (West and Gundersen 1990; West et al. 1991) on approximately 10 equidistant sections covering the full anteroposterior range of the projection, except for one case injected with BDA in area 21a in which sampling was done on 5 sections. Axonal swellings were then counted in square disectors that were 15 × 15 μm and 15 μm height. Care was taken to avoid sampling in the 2–3 μm immediately adjacent to the sections surfaces to avoid measuring cut or damaged terminals. The maximum diameter was measured for each sampled swelling. This optical fractionator sampling strategy allowed for the estimation of the total number of swellings in the pulvinar. The total numbers of swellings (*N*) were calculated using the following equation provided by (West et al. 1991).

$$ N=\sum Q\times{ssf}^{-1}\times{asf}^{-1}\times{tsf}^{-1} $$$$

where Σ*Q* is the total number of swellings counted within the disectors, and *ssf* is the section sampling fraction (number of sampled sections over the total number of sections on which the terminal projection field appears); *asf* is the area sampling fraction (ratio of the frame area/the total area of the reference space on the section), and *tsf* is the thickness-sampling fraction (disector height/measured section thickness). The overall sampling faction (see Table 1) is the product of *ssf*, *asf,* and *tsf*. Coefficients of error were calculated (West and Gundersen 1990; West et al. 1991) in order to determine whether the sampling effort was sufficient. It is widely accepted as a rule of thumb that coefficients of error below 0.1 are indicative of a sufficient sampling (West and Gundersen 1990). Stereological sampling parameters and number of objects counted for every case are shown in Table 1. For injections made in area 21a, all sections used for systematic stereological sampling of boutons in the thalamus were also carefully scanned at high magnification (40 and 100×) for the mapping of rare objects (rosette-like terminals and singleton), which could have been underestimated by the optical fractionator workflow.

**Table 1:** Stereological sampling parameters for the estimation of the number of anterogradely labeled axonal swellings in each subdivision of the LP pulvinar after BDA injections in area 21a and 17

Case	Region	Number of sections	Total area (μm^2^)	Number of disectors	Number of objects counted	Sampling fraction	Total estimation (*N*)	CE (*N*)	CE (*Q*)
A21 a1	LPl	15	5 634 351	302	798	0.007	113 076	0.11	0.05
	LPm	10	4 128 436	209	801	0.007	122 067	0.08	0.03
A21a 2	LPl	18	4 975 310	259	528	0.006	91 347	0.10	0.05
	LPm	13	4 561 028	240	464	0.006	81 874	0.10	0.04
A21a 3	LPl	5	1 763 878	100	240	0.010	22 891	0.16	0.10
	LPm	6	1 504 564	84	157	0.010	15 449	0.11	0.07
A21a 4	LPl	6	3 025 878	161	349	0.007	47 777	0.14	0.08
	LPm	11	4 046 560	221	369	0.008	47 717	0.05	0.09
A17	LPl	10	2 044 291	110	333	0.008	41 372	0.05	0.02

The criteria used for the morphological identification and classification of the axon terminals were those of (Guillery et al. 2001). Type I CT axons were thin, possessed small caliber beaded terminals on the axon itself classified as *en passant* or as single boutons at the end of short side branches classified as thin short stalks; while type II axons possessed thicker axons terminating in one or several small compact arbors showing complex arborization. Terminals on type II axons identified as singleton, that is, single beaded axon endings or rosette-like terminals for complex cluster of more than 3 terminals. For comparison purpose, previous cases from area 17, PMLS cortex and the AES projections were reconsidered (Huppe-Gourgues et al. 2006; Huppe-Gourgues et al. 2019).

### Statistical Analyses

Statistical analyses were performed using SPSS v 16.0 software (SPSS, Chicago, IL, USA). Differences in the proportions of projection types from both sub nuclei in each and across all animals were compared with the Wald Khi-square test. Differences observed in the size distribution of boutons from areas 17 and 21a projections to the LPl and LPm were compared with the 2 sample Kolmogorov–Smirnov test. The association between the hierarchical rank of visual areas and the increasing number of type I projection to the pulvinar was tested using Spearman rank correlation coefficient. All tests were performed with a significance level of *P* < 0.05.

## Results

### Injection Sites in Area 21a and 17

**Figure 1:**
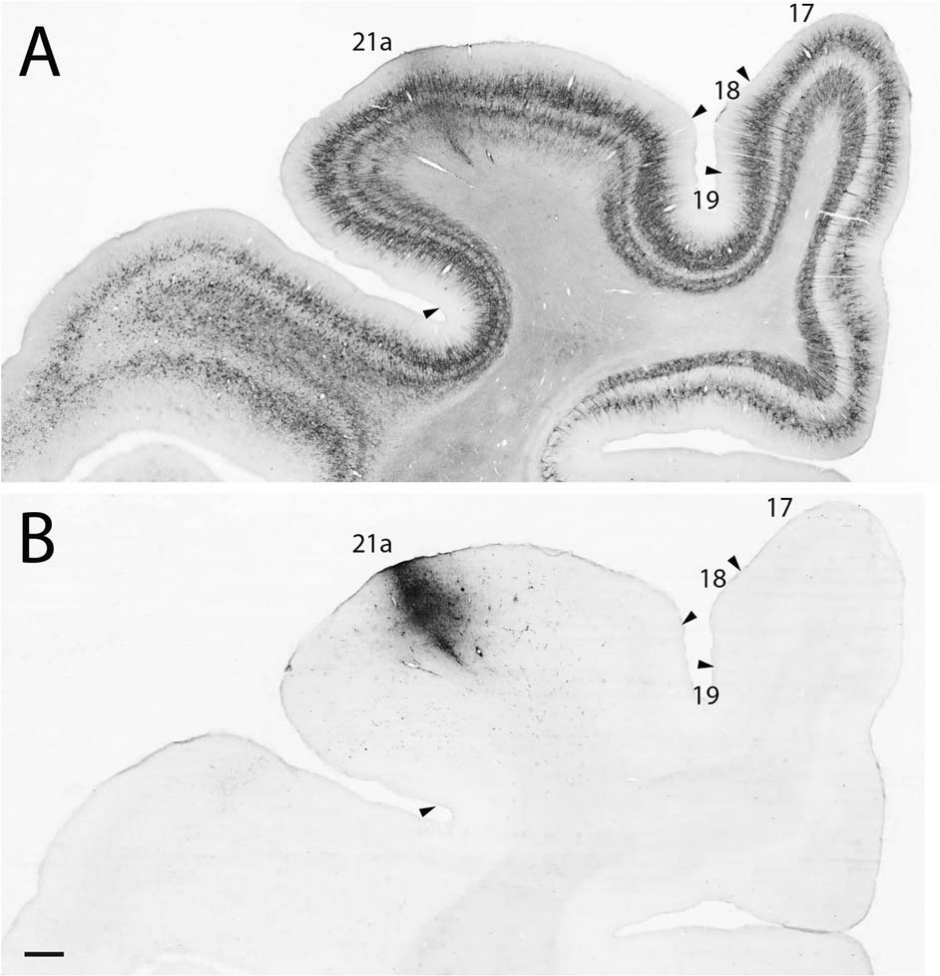
Low power photomicrographs of (*A*) SMI-32 immunostained coronal section showing localization of area 21a and surrounding visual cortical areas (from case A21a 3). (*B*) The localization of a BDA injection in area 21a. Scale 500 μm.

All injections in area 21a were aimed at its crown to avoid spilling over in adjacent area 19 (Fig. 1*A* and Supplementary Fig. 1). The injection in area 17 also avoided adjacent area 18 by targeting its medio-posterior part (Supplementary Fig. 1*D*). Moreover, because type I projections arise from layer VI while type II from layer V (Ojima 1994; Bourassa and Deschênes 1995), all injections were performed at 2 depths (0.8 and 1.2 mm), with an angle perpendicular to the cortical surface. Careful inspection of injection sites confirmed the targeting of all layers, but layer V and VI in particular, while no contamination of the white matter was observed. SMI-32 staining patterns (Fig. 1*A*) on adjacent sections confirm the localization of the injection sites (Fig. 1*B*). An example of an injection site centered on the suprasylvian gyrus and targeting all layers of cortical area 21a can be seen in (Fig. 1*A*). SMI 32 staining patterns on adjacent sections confirm the localization of the injection sites (Fig. 1*B*).

### Area 21a and 17 Projection Sites in the LP-pulvinar

**Figure 2:**
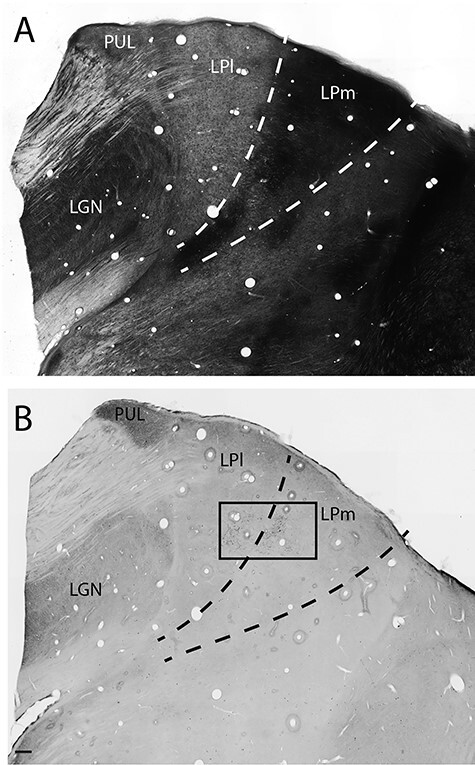
Low power photomicrographs of coronal sections of the thalamus stained for AChE (*A*) showing the dark staining in LPm compared with LPl and BDA histochemistry showing the terminal fields of anterogradely labeled cortical projections from area 21 (*B*). Scale 250 μm.

**Figure 3:**
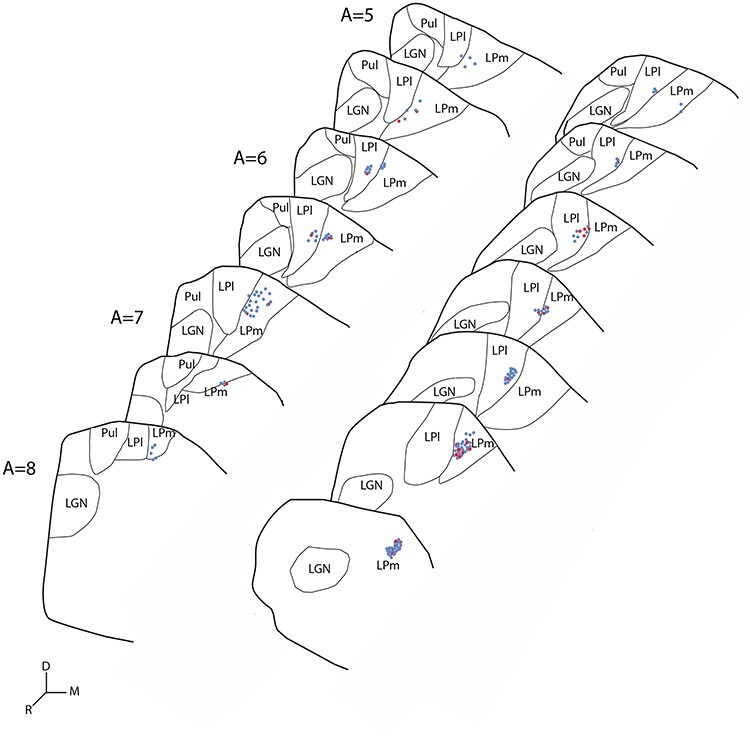
Schematic representation of the distribution of stereologically estimated BDA labeled terminals in coronal sections of the thalamus of 2 cases showing projections from area 21a to the LP in gray. Blue dots: type I axon terminals, Red dots: type II axon terminals, one dot represents 5 terminals, Scale 1 mm.

AChE histochemistry was used to distinguish the medial (LPm) and lateral (LPl) divisions of the LP (Fig. 2*A*). Significant labeling of cortical terminals in the LP-Pulvinar complex was observed following injections in area 21a (Fig. 2*B*). Cortical projections were observed in both the LPl, as well as in the LPm, the striate- and tecto-recipient zones of the LP, respectively (Fig. 2*B*). While some projection foci were strictly located in the LPl or the LPm, some were seen targeting their border and extending in both subnuclei. Most projections observed were also spatially restricted to confined foci suggesting a relatively precise topographic organization. In all cases, several distinct foci of projections were observed within one subdivision of the LP, each one spreading over several sections (Fig. 3). Most projection foci in both nuclei were loosely organized in a columnar manner that runs from the ventral to the lateral tip of the LP in an oblique manner (Fig. 2*B*).

Significant labeling of CT axons was also observed following injection in area 17 but in contrast to area 21a, labeled terminals were confined in a single focus in the more ventral and caudal portions of the Lateral LP nucleus, immediately medial to the dLGN. This region corresponds to the striate recipient zone of the LP as originally described by (Graybiel and Berson 1980) or the LPL 1 described by (Chalupa et al. 1983).

### Classification of Anterogradely Labeled Axons from Area 21a and Area 17

**Figure 4:**
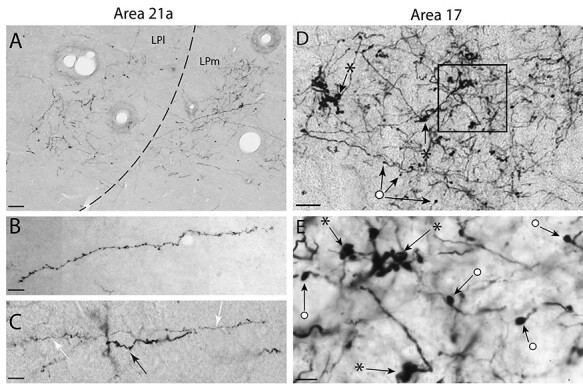
(*A*) High-power photomicrograph from the boxed area of Figure 2*B* showing projection foci of anterogradely labeled CT axon terminals in the LPl and the LPm from area 21a. (*B*) High-power photomicrograph showing CT axons from area 21a exhibiting a typical type I morphology with (thin) short stalks and occasional (thin) *en passant* boutons. (*C*) A typical type I axon (white arrow) alongside a thicker axon (black arrow) with a similar morphology of (thicker) short stalks and occasional (thicker) *en passant* swellings on the axon itself classified as type II. (*D*) Projection foci of CT axons from area 17 in the LPl displaying complex arborization with several terminals formed of multiple large boutons in a rosette-like structure (stars) as well as singletons which are large single swellings at the end of axonal side branches or *en passant* boutons (circles). (*E*) Magnified high-power photomicrograph from the boxed area of panel D displaying singletons (circles) and rosette (stars) terminals. Scales: (*A*) 50 μm; (*B* and *C*) 25 μm; (*D*) 40 μm; (*E*) 10 μm.

Following the localization of projection foci in the LP Pulvinar complex originating from area 17 and 21a, axons in both sites were studied and classified according to their morphology. An example of a CT projection focus from area 21a to the pulvinar complex is shown in Figure 4*A*, where a high magnification photomicrograph reveals the morphology of terminals and their overall axonal branching pattern. The vast majority of axons observed in the pulvinar complex were of thin caliber (Table 2), giving rise to small boutons that emanated from thin side branches that were classified as “thin short stalks”. Swellings on the axons were classified as “thin en passant”. These axons were linear and poorly branched (Fig. 4*A*–*C*). Using the nomenclature of Guillery et al. 2001, these projections were classified as type I. A typical CT type I axon with both *en passant* and short stalk boutons is presented in panel B. In some cases, thicker axons were observed traveling among thinner type I projections (Fig. 4*C*) and they were classified as type II. They also had either short stalk boutons (classified as “thick short stalk”) or *en passant* boutons (classified as “thick en passant”).

**Table 2:** Percentage of CT terminal types following injections in area 21a in the LPl and LPm

%		Subdivision of the LP pulvinar	
		LPI	LPM
A21a 1			
Type I	Thin *en passant* Thin short stalk Total	57 (452) 14 (115) 71 (567)	65 (521) 6 (50) 71 (571)
Type II	Thick *en passant* Thick short stalk Singleton/rosette Total	15 (119) 13 (107) 1 (5) 29 (231)	16 (126) 13 (101) 0 (3) 29 (230)
A21a 2			
Type I	Thin *en passant* Thin short stalk Total	66 (349) 21 (112) 87 (461)	63 (292) 19 (88) 82 (380)
Type II	Thick *en passant* Thick short stalk Singleton/rosette Total	5 (25) 8 (41) 0 (1) 13 (67)	8 (39) 10 (45) 0 (0) 18 (54)
A21a 3			
Type I	Thin *en passant* Thin short stalk Total	55 (131) 28 (67) 83 (198)	71 (111) 15 (24) 86 (135)
Type II	Thick *en passant* Thick short stalk Singleton/rosette Total	10 (25) 7 (16) 0 (1) 17 (42)	11 (18) 3 (4) 0 (0) 14 (22)
A11a 4			
Type I	Thin *en passant* Thin short stalk Total	61 (214) 30 (103) 91 (317)	57 (212) 34 (124) 91 (337)
Type II	Thick *en passant* Thick short stalk Singleton/rosette Total	2 (8) 7 (23) 0 (1) 9 (32)	3 (11) 5 (19) 1 (3) 9 (33)
Mean A21a for all cases			
Type I	Thin *en passant* Thin short stalk Total	60 (1146) 21 (397) 81 (1543)	63 (1136) 16 (286) 79 (1422)
Type II	Thick *en passant* Thick short stalk Singleton/rosette Total	9 (177) 10 (187) 0 (8) 19 (372)	11 (194) 10 (169) 0 (6) 21 (369)

In contrast with CT axons from area 21a, projections from area 17 displayed a more complex arborization and a wide variety of bouton morphologies and sizes (Fig. 4*D*). Moreover, unlike projections from area 21a, which mostly exhibited typical type I thin caliber axons, type I inputs were less numerous following area 17 injections (25%). Also, while large type II rosette-like structures were not observed, and that large singleton boutons were scarce in the projection originating from area 21a, these terminal morphologies were frequently observed following injections in area 17 (Fig. 4*D*,*E*).

### Quantitative Analysis

#### Proportion and Frequency Distribution of Terminals from Area 17 and Area 21a

**Figure 5:**
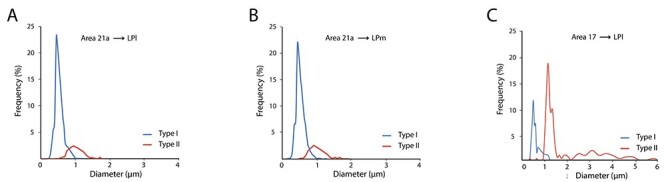
Frequency distribution of CT bouton diameter from area 21a in the LPl (*A*), the LPm (*B*), and from area 17 to the LPl for type I (blue) and type II (red) terminals.

To better compare the contribution of CT projections from areas 21a and 17, every bouton sampled in the pulvinar complex was measured and classified according to its morphology for each LP subdivisions and for both cortical areas. As expected for area 21a, type I boutons represented the vast majority (81%) of boutons counted in the LPl, (Table 2) significantly outnumbering type II boutons (19%) (Wald Khi-square test, *P* < 0.001); type I boutons were found to be significantly smaller than boutons from type II axons (Fig. 5*A*). The average bouton size from type I CT axons originating from area 21a in the LPl was 0.49 μm (±0.01) and 0.99 μm (±0.03) from type II axons. Both size distributions differed significantly (Kolmogorov–Smirnov test, *P* < 0.001).

In the medial part of the LP (Fig. 5*B*), a similar size distribution and proportion of axonal types were observed following injections in area 21a. Indeed, type I boutons (79%) (Table 2) significantly outnumbered those from thicker type II axons (21%) (Wald chi-square test, *P* < 0.001). Similarly, in the LPl the average bouton diameter of type I (0.49 μm, ±0.01) axons was also smaller than that of type II axons (1.00 μm; ±0.04). Both size distributions differed significantly (Kolmogorov–Smirnov test, *P* < 0.001).

Both subdivisions of the LP have different anatomical and functional connectivity patterns: in essence, the LPl is the only recipient zone of striate projections and the LPm, the most prominent recipient zone of collicular input (a small zone in the LPl, named LPl2, received collicular inputs (Abramson and Chalupa 1985). Area 21a, however, projects to both subdivisions. We thus investigated whether the strength and distribution profile of area 21a projections to the 2 subnuclei differed by comparing the estimated number of boutons (*N*), the proportion of type I and type II boutons, and their size distribution. Even though the number of labeled neurons varied between cases and is dependent upon injection size, for each case, all axons labeled in the LPl and LPm originated from the same injection and therefore, the estimated number of boutons (*N*) in both subdivisions of the LP is instructive of the strength of the projection. The average number of estimated objects *N* was 68 772 for the LPl and 66 776 for the LPm. No difference was observed in the strength of these projections from area 21a to both subdivisions of the LP (Student’s *t*-test *P* = 0.33). No difference was also observed in the proportion of type I versus type II boutons in the LPl and LPm (Wald chi-square test, *P* = 0.976). The average size distribution of sampled boutons in both subnuclei was also compared: in all cases, no difference was seen between the size distribution of counted boutons in both subdivisions. The average size was 0.59 (±0.01) and 0.60 μm (±0.01) for the LPl and LPm**.** Both size distributions did not differ in a significant manner (Kolmogorov–Smirnov test, *P* = 0.189).

**Figure 6:**
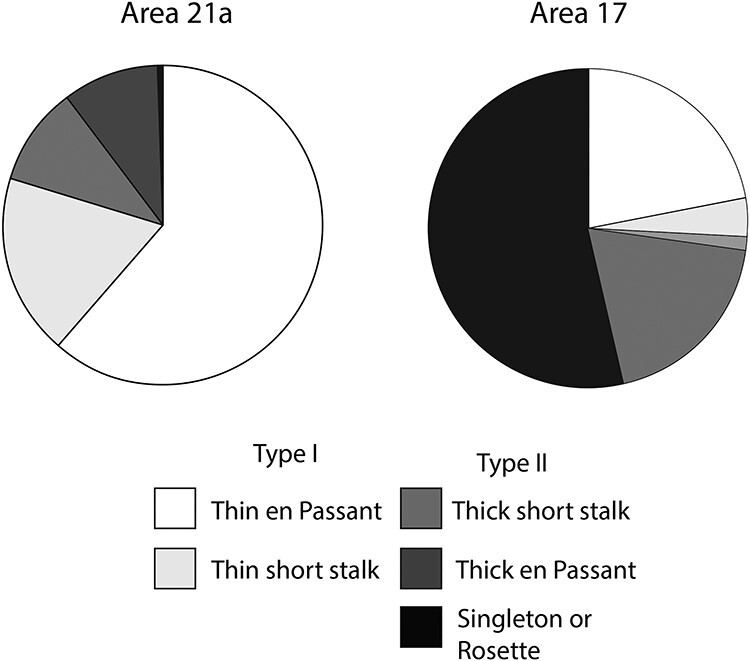
Pie chart representation of % of bouton types for area 21a and area 17.

The frequency distribution of bouton’s largest diameter was also plotted for projections originating from area 17 (Fig. 5*C*). Unlike boutons from area 21a whose size was mostly found to be below 1 μm, a significant number of boutons from area 17 were larger than 1 μm, many of them having a diameter greater than 3 μm. Type II terminals were again found to be larger than type I terminals. The average bouton diameter for type I was 0.46 μm (±0.01) and 1.44 μm (±0.07) for type II terminals. When the average bouton diameter was compared between CT axons from area 17 and 21a, average bouton diameter from area 17 were found to be greater (1.04 μm ± 0.04) than area 21a terminals (0.6 μm ± 0.01). The size distribution of boutons from area 17 and 21a was significantly different (Kolmogorov–smirnov test, *P* < 0.001). When compared with projections from area 21a, which were mostly of type I (80%), the latter represented only a fraction (25%) of the total projections coming from area 17 (Fig. 6).

### Type I Terminals Increase as a Function of Cortical Hierarchy

**Figure 7:**
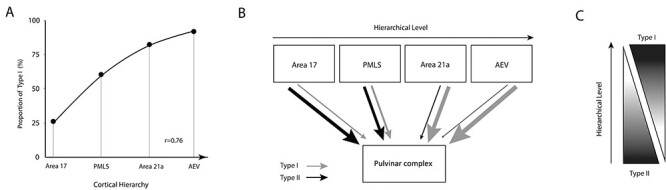
(*A*) Proportion in % of type I terminals as a function of the cortical hierarchy (data from the PMLS and the AEV were reconsidered from Huppe-Gourgues et al. 2019). (*B*) Schematic summary of CT circuitry involving the pulvinar where the thickness of gray and black arrows represents the proportion of type I and type II projections, respectively. (*C*) A general schematic of the changing proportion of type I and type II CT projections as a function of cortical hierarchy.

To test the hypothesis that CT projections from the cortex to the pulvinar varies as a function of cortical hierarchical levels, the proportion of type I terminals sent by various cortical areas was plotted as a function of the hierarchy of the cat cortical areas (Fig. 7) (Scannell et al. 1995).

Data from the present study were compared with those obtained in our laboratory using the same experimental approaches (Table 3) (Huppe-Gourgues et al. 2019). The proportion of type I projection increases as one ascends along the visual cortical hierarchy, with type I proportion being around 25%, 66%, 82% and 91% for area 17, PMLS, area 21a and the AES projections, respectively (Fig. 7*A*). There was a strong positive correlation (*r* = 0.76) between the proportion of type I terminals and the assumed cortical hierarchy (Spearman’s rank correlation, *P* = 0.002).

**Table 3:** Percentage of CT terminal types according to cortical origin for 4 hierarchically organized areas (area 17, PMLS, area 21a, and AES)

%		LPI	LPm
Area 17	Type I Type II	25 75	
Area PMLS	Type I Type II	71 29	72 28
Area 21a	Type I Type II	81 19	79 21
AEV	Type I Type II		91 9

## Discussion

The present results indicate that the majority of CT axons from area 21a projecting to each subdivision of the LP-pulvinar complex exhibit type I terminals. Most importantly, our results demonstrate that the proportion of modulatory CT inputs increases with the hierarchical level of the visual areas studied. These results are a unique anatomical demonstration that the organization of transthalamic pathways reflects the cortical hierarchy. They provide a new framework for a better understanding of the organization and role of transthalamic cortical pathways as well as for the establishment of more accurate models of visual processing along the transthalamic cortical routes.

### Topographic Organization of CT Projections

We found projection foci in both subdivisions of the LP that spread over several sections (Hutchins and Updyke 1989). Other tracing studies using similar protocols also reported that injections made in area 17, 18, 19, the PMLS, areas 5 and 7 resulted in distinct projections patches spread over several sections (Guillery et al. 2001; Baldauf et al. 2005; Huppe-Gourgues et al. 2006). Our observation suggests that retinotopically specific zones of area 21a are likely to be represented several times in the LP, in line with the multiple visual field representations described in electrophysiological studies (Raczkowski and Rosenquist 1981; Updyke 1983).

### Morphology of Area 21 Projections to the Pulvinar

We show here that most axons originating from area 21a were type I axons presenting beaded terminals linked by a small stalk and small swellings on fine caliber axons. Thicker type II axons were also found with morphologies and boutons resembling those of type I but only bigger. In a previous study (Abramson and Chalupa 1985) found that area 21a sends projections to both LP subnuclei in a similar fashion and that these axons arise from layer V and VI. While they do not report quantitative data regarding the cortical layer of origin of these cortico-pulvinar inputs, they mention that projections from layer VI outnumbered those of layer V in extrastriate areas including area 21a. Other studies have also confirmed the existence of 2 distinct laminar origins of CT projection in the visual system and other sensory and motor systems in several animal models (Ojima 1994; Bourassa and Deschênes 1995; Rockland 1996; Rouiller et al. 1998; Darian-Smith et al. 1999). They all support the observation that projections from layer VI to HO thalamic nuclei bear Type I projections, while those from layer V are of type II. Therefore, results from Abramson and Chalupa (1985), which reported a greater number CT cells from XC areas including area 21a originating from layer VI than layer V are in line with our observation of a greater proportion of type I terminals in the pulvinar while further strengthening the view that type I and II CT projections arise from layer VI and V, respectively.

We did not find any significant difference between the strength, bouton size and proportion of terminal types of area 21a projection to the 2 subdivisions of the LP nucleus, the LPl and LPm. These results are in line with prior qualitative observations made by Abramson and Chalupa (1985), which reported that cortical areas beyond areas 17 and 18 had a similar organizational projection patterns, irrespective of their thalamic target (tecto- or striate-recipient zones of LP).

### Functional Significance of Type I and Type II Projections

The existence of 2 types of CT projections in the pulvinar and other HO thalamic nuclei has been demonstrated in several anatomical studies (Bourassa and Deschênes 1995; Rockland 1996; Rouiller and Welker 2000; Guillery et al. 2001). While few of these studies have investigated the functional role of the CT projections, there is evidence that the “driver and modulator” function attributed respectively to type II and type I terminals along the retino-geniculo-cortical route may be generalized to extrageniculate pathways.

For instance, most projections from the primary visual cortex to LP-pulvinar complex have type II terminals (Rockland 1996; Feig and Harting 1998; Guillery et al. 2001; Huppe-Gourgues et al. 2019). According to the “driver/modulator” framework proposed by Sherman and Guillery, these type II projections from V1 should have a driver-like influence on pulvinar neurons and therefore be critical for the establishment of their receptive field properties. Electrophysiological data confirm this assumption since the deactivation of area 17 decreases receptive field responsiveness of neurons in the pulvinar of mice, cats, and primates (Casanova et al. 1997; Rushmore et al. 2005; Bennett et al. 2019; Bender 1983), suggesting a common ground of functional organization among species.

We expect that, CT projections from area 21a, which mainly arise from layer VI neurons, will have a different functional role than those coming from layer V area 17 neurons, thus supporting the view that corticofugal pathways originating from layers V and VI have distinct influences on thalamic activity (Li et al. 2003; Van Horn and Sherman 2004; Usrey and Sherman 2019). To our knowledge, no study investigated the impact of deactivating area 21a (or V4 in primates) on the visual responsiveness of LP-pulvinar neurons. Additional physiological experiments are thus required to determine whether this pathway carries modulatory signals. The fact that the pulvinar complex deactivation has different functional effects on visual responses of area 17 and 21a (de Souza et al. 2019) may suggest that, in return, the deactivation of both these areas would have a different effect on visual responses of pulvinar neurons.

### CT Axon Morphology Differs in Striate and Extra Striate Areas

Our results indicate that the proportion of terminal types varies between striate and extrastriate areas. While only qualitative, observations reported by Guillery et al. (2001) also indicate that the bouton morphology of CT axons originating from extrastriate area 19 differs to those from areas 17 or 18. A similar trend was also observed for corticopulvinar axons from areas 5 and 7 (Baldauf et al. 2005). Variations in the morphology of striate and extrastriate corticopulvinar terminals have also been qualitatively observed in other species such as gray squirrels (Robson and Hall 1977), macaques (Rockland 1996), and tree shrews (Chomsung et al. 2010; Day-Brown et al. 2017), suggesting a common pattern of CT projection among species.

### Toward a Hierarchical Organization of CT Input

A fundamental question that remains to be answered in the context of cortical communication is whether transthalamic pathways are organized in a hierarchical manner. Our results from area 21a, when added to those obtained from the PMLS and the AES reveal the existence of a positive correlation between the percentage of type I terminals and the level of cortical hierarchy such that the proportion of type I terminals increases as one travels along the hierarchy of visual areas. These results therefore demonstrate that, like cortico-cortical connections (Salin and Bullier 1995; Scannell et al. 1995; Markov et al. 2014), CT projections also follow a hierarchical model when projecting to HO thalamic areas.

Most importantly, there is some evidence that this organization may be present in other species. Even if these reports were only qualitative. Anatomical observation in the gray squirrel (Robson and Hall 1977), tree shrew (Day-Brown et al. 2017) (Chomsung et al. 2010), and macaque monkey (Rockland 1996) has shown that type I CT projection to the pulvinar may be more dominant from higher visual areas than those arising from the striate cortex. Thus, the hierarchical organization of CT projection in the visual system may very well be applicable to species other than the cat. There are also some indications suggesting a common organizational framework across sensory systems. First, according to our data, the projection pattern of CT projections in the visual system is based upon the hierarchical organization of cortical visual areas (Scannell et al. 1995). This suggests that CT projections are organized in a way that reflects the organization of their cortico-cortical counterparts. In this context, it is important to mention that other sensory systems, such as the auditory and somatosensory ones, are also organized in a hierarchical manner (Iwamura 1998; Kaas and Hackett 2000; Whitehead et al. 2019). Moreover, the classification of transthalamic CT projections into type I and type II morphologies has also been reported in other sensory systems where they also emanate from layer VI and V, respectively (Rouiller and Welker 2000). This suggests the existence of a common framework for the organization of these projections across both species and systems.

The fact that CT projection pattern follows the anatomical hierarchical organization of cortical areas brings important functional implication. Should the organization of CT projections be built upon that of cortico-cortical ones, one may also expect to find, within the pulvinar, an internal organization that would reflect the existence of dorsal and ventral functional pathways. In the macaque, evidence suggests that the anatomical connectivity pattern of specific subnuclei of the pulvinar show a bias toward areas of the ventral or dorsal stream (Kaas and Lyon 2007; Kaas and Baldwin 2019), an organization which may also be present in the mouse (Bennett et al. 2019) further strengthening the emerging view that fundamental units of CT computations are not individual thalamic nuclei but more precise thalamic networks linking functionally related cortical areas (Shipp 2003; Halassa and Kastner 2017).

## Conclusion

Results from this study provided new information regarding the organization of CT projections from striate and extrastriate areas of the visual system. Most importantly, our results demonstrate that the proportion of modulatory CT inputs increases with the hierarchical level of the visual areas. These results are the first anatomical demonstration that the organization of transthalamic pathways reflects cortical hierarchy providing the basis for the establishment of more accurate models of CT flow of information in the visual system and potentially in other sensory systems and species.

## Notes

*Conflict of Interest*: None declared.

## Funding

Canadian Institute of Health Research (grant PJT-148959 to C.C.); Natural Sciences and Engineering Research Council of Canada Discovery (grant RGPIN-203702 to D.B.). R.A.F. received financial support through the Ezell fellowship by the American Academy of Optometry Foundation for his doctoral studies.

## Supplementary Material

Suppl_Fig_1_tgaa030
