# Supplementary figures and images for: Hierarchical Organization of Corticothalamic Projections to the Pulvinar
Source: Cereb Cortex Commun. 2020 Jul 7;1(1):tgaa030. doi: 10.1093/texcom/tgaa030 (PMC8152833; doi:10.1093/texcom/tgaa030)

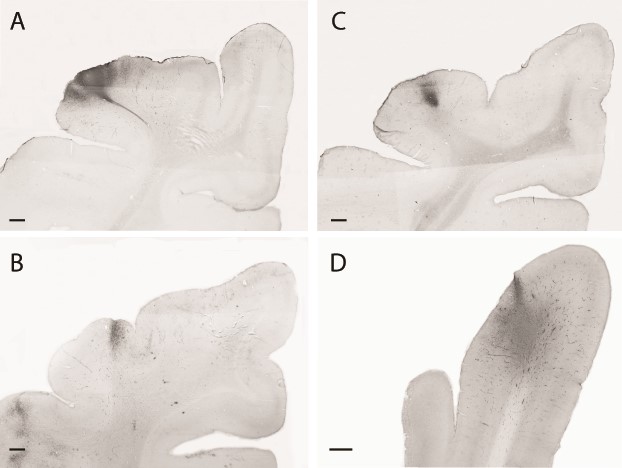

Supplement: Suppl_Fig_1_tgaa030 [file suppl_fig_1_tgaa030.jpeg]
